# Ketamine Sedation in Gastrointestinal Endoscopy in Children

**DOI:** 10.3889/oamjms.2016.085

**Published:** 2016-07-26

**Authors:** Ayman E. Eskander, Nevine R. El Baroudy, Amira S. El Refay

**Affiliations:** 1 *Department of Pediatrics, Faculty of Medicine, Cairo University, Cairo, Egypt*; 2 *Departments of Child Health, National Research Centre, Cairo, Egypt (Affiliation ID: 60014618)*

**Keywords:** Pediatric endoscopy, sedation, Ketamine, safety, complications

## Abstract

**BACKGROUND:**

Moderate sedation for gastrointestinal endoscopy has traditionally been provided by the endoscopist. Controversy has ensued over safe and efficient sedation practice as endoscopy has increased in numbers and complexity.

**AIM:**

To evaluate the safety of ketamine sedation given by non-anesthesiologist during gastrointestinal endoscopy in children.

**METHODS:**

A prospective study of 100 paediatric patients with gastrointestinal symptoms who were a candidate for upper or lower gastrointestinal endoscopy in paediatric endoscopy unit at Abo El-Reesh Paediatric Hospital, Cairo University. All children were > 2 years old and weighed > 6 kg. The analysis was performed in terms of sedation-related complications.

**RESULTS:**

A total 100 paediatric patients including 53 males and 47 females with mean age of 5.04 years were involved in the study. All children were medicated with ketamine with a mean dose of 3.77mg/kg. No complications occurred in 87% of cases. Desaturation occurred in 13% of the cases and was reversible by supplemental nasal oxygen. Desaturation was more frequent during Upper GI Endoscopy and with the intramuscular route (p value=0.049). No apnea, bradycardia, arrest or emergence reactions were recorded.

**CONCLUSION:**

Ketamine sedation found to be safe for paediatric gastrointestinal endoscopy in Egyptian children without co-morbidities. Transient Hypoxia (13%) may occur but easily reversed by nasal oxygen therapy.

## Introduction

Although gastrointestinal endoscopy is widely accepted as fundamental to the diagnosis and treatment of digestive disorders in children, considerable controversy and practice differences persist with respect to the methods and agents used to achieve optimal endoscopic sedation [1]. Sedation must have a rapid onset, short duration of action, and should be safely administered by a non-anesthesiologist without significantly increased the risk of potential complications [2].

Ketamine is a general anaesthetic agent widely used for paediatric procedural sedation outside the operating theatre by non-anesthesiologists [3]. It is considered a dissociative anaesthetic. This means that the drug distorts the user’s perception of sight and sound and produces feelings of detachment from the environment and one’s self [4]. Ketamine has found many applications in paediatric anaesthetic practice. Insights into the mechanism of action and the pharmacokinetics and pharmacodynamics of its isomers have led to a re-evaluation of this drug, expanding the range of applications in children. Ketamine is a remarkably versatile drug that can be administered through almost any route. It can also be used for different purposes [5].

Prior studies of ketamine sedation for paediatric gastrointestinal endoscopy have been retrospective in nature and have used chart review to identify any clinical concerns for inadequate sedation [6, 7].

The aim of the present study was to evaluate the safety of ketamine sedation given by non-anesthesiologist during upper and lower gastrointestinal endoscopy in children. Also, this study aims to detect possible complications from sedation in the upper and lower gastrointestinal endoscopy in children and relation between the disease history and clinical condition of the patient with possible complications of sedation by ketamine.

## Patients and Methods

This prospective study was conducted upon 100 paediatric patients with gastrointestinal symptoms who were a candidate for upper or lower gastrointestinal endoscopy presented to Pediatric Endoscopy Unit, Abo El-Reesh Pediatric Hospital, Cairo University.

All patients had the appropriate instructions for gastrointestinal endoscopy according to its type.

The study followed the regulations of the medical ethical committee of Abo El-Reesh Pediatric Hospital, Cairo University and written Informed consent was obtained from the appropriately designated parent or guardian.

A full and detailed history was taken from one of the parents or guardians regarding the disease, nutritional and drug history including drug allergies.

A full clinical examination of the patient was done including general, cardiac, chest and abdominal examination.

Pre-sedation risk assessment of cardiopulmonary status including heart rate, respiratory rate, oxygen saturation and blood pressure measurement was done before ketamine administration.

### Ketamine Administration

- Intravenous (IV) Route: A loading dose of 1.5 to 2 mg/kg was administrated. The procedure started 30 seconds to 1 minute after administration. Additional incremental doses of ketamine may be administered (0.5 to 1 mg/kg) if initial sedation was inadequate or to accomplish a longer procedure.
- Intramuscular (IM) Route: Ketamine was administrated with a dose of 3 to 5 mg/kg.

Clinical onset began within 5 minutes. Duration of effective dissociation was 20 to 30 minutes.

Sedation was actively administered and supervised by a paediatrician. Procedures were performed by four different endoscopists and also attended by two nurses educated in sedation pharmacology and gastrointestinal endoscopy.

Constant patient monitoring during and after the administration of ketamine was provided including continuous cardiac monitoring, respiratory rate, pulse oximetry and blood pressure monitoring.

Sedation-related complications include a drop in oxygen saturation to equal or less than 94%, respiratory distress (stridor or wheezes), apnea, bradycardia (< 60 beats per minute), cardiac arrest, emergency reactions and need for reversal medication were observed. All patients were put under observation for 2 hours after the procedure to observe any complications.

### Statistical analysis

Statistical analysis was performed using the SPSS statistical package software for windows version 21 (SSPS Inc, Pennsylvania, USA). Parametric variables are expressed as the mean ± SD. P value <0.05 was considered significant.

## Results

The patients included 53 males (53%) and 47 females (47%). Their age ranged from 2 to 12 years with a mean of 5.04 years, while their weight ranged from 6 to 36 kg with a mean of 16.92 kg and they received ketamine with a dose ranged from 2 to 5 mg/kg with a mean of 3.77 mg/kg (Table 1).

**Table 1:** Ketamine doses in studied cases

	Mean ± SD	Median	Minimum	Maximum
Age (years)	5.04 ± 3.120	4.50	2	12
Weight (kg)	16.92 ±7.388	15.00	6	36
Ketamine dose (mg/kg)	3.77 ± 0.851	4.00	2	5

Most of the studied cases (42%) were indicated for GI endoscopy for diagnostic purposes, while 24 cases (24%) were done for follow-up of oesophagal varies, 1 case (1%) was done routinely before renal transplantation (Table 2).

**Table 2:** Indications of endoscope in studied cases

	Frequency(No)	Percent (%)
Dilatation	23	23
Diagnostic	42	42
Foreign body extraction	10	10
Follow-up of oesophagal varices	24	24
Routine before renal transplantation	1	1
Total	100	100

In pre-sedation assessment and examination, we found 3 cases with tachycardia and 4 cases with hypertension for age, 2 cases with cardiac murmur, 4 cases with wheezy chest and respiratory distress, 5 cases with ascites and there was no significant relation between these findings and occurrence of complications except for those who had respiratory distress, all of them developed hypoxia during the procedure (p value=0.001)

The only complication that occurred in our study was desaturation; there were 13 patients who developed hypoxia during the procedure. These patients developed a decrease in oxygen saturation to less than 94% by pulse oximetry. These attacks of hypoxia were easily reversed by supplemental nasal oxygen.

In our study, Cases are classified into four groups according to the type of the procedure performed (Upper GI endoscopy, Colonoscopy) and the route of ketamine administration (IM, IV)

Group (1) had upper GI endoscopy and received ketamine intravenously. Group (2) had upper GI endoscopy and received ketamine intramuscularly. Group (3) had a colonoscopy and received ketamine intravenously. Group (4) had a colonoscopy and received ketamine intramuscularly.

There was a significant relation between the route of ketamine administration and type of performed procedure with occurrence of complications, hypoxia occurred during Upper GI Endoscopy (14.28%) more than that occurred during lower GI Endoscopy (8.69%) and with the intramuscular administration of ketamine (15.38%) more than the intravenous (8.57%) (p value=0.49), (Table 3).

**Table 3:** Classification of studied cases according to the type of the procedure performed (Upper GI endoscopy, Colonoscopy) and the route of ketamine administration (IM, IV)

	Group 1		Group 2		Group 3		Group 4		P value
	NO	%	NO	%	NO	%	NO	%	
Hypoxia	3	11.1	8	16	0	0	2	13.3	0.049
Normal	24	88.9	42	84	8	100	13	86.7	

There was no relation between ketamine dose and occurrence of complications. After the procedure, one of the studied cases had an attack of vomiting that didn’t recur.

## Discussion

This prospective study was designed to detect complications of ketamine sedation given by non-anesthesiologist in gastrointestinal endoscopy in children. A total of 100 cases presented to the Gastrointestinal Endoscopy Unit of Abo El-Reesh Pediatric hospital.

In our study, there were 13 patients who developed hypoxia during the procedure and it was reversed by supplemental oxygen. These patients developed a decrease in oxygen saturation to less than 94% by a pulse oximeter. Most of these patients developed desaturation within few minutes from the introduction of the endoscopy. This desaturation is thought to be due to laryngospasm. Although ketamine has been deemed safe based on it’s cardiovascular and respiratory protective effect [6, 7] it is also associated with increased risk of laryngospasm [9, 10].

Most of the episodes of desaturation occurred during Upper GI Endoscopy (14.28%), where stimulation of the posterior pharynx may increase the risk of airway complications. Desaturation also occurred more frequent with the Intramuscular route (15.38%), so Desaturation was more frequent in the group (2) (16%) than group (1) (11.1%).

All patients who had respiratory distress by examination before injection of ketamine had hypoxia during the procedure (p value=0.001).

The study was done by Green and Johnson, 1990 [11] of 11,589 patients, the incidence of laryngospasm was 0.4%, and that when it did occur, it was easily treated, only two cases of ketamine-associated laryngospasm led to intubation.

Unlike to our study, the incidence of desaturation less than 94% was 13%, but destruction may be due to laryngospasm or other causes related (Increased bronchial secretions) or not related to ketamine sedation such as associated chest infection as all patients who had respiratory distress and wheezy chest by examination before injection of ketamine developed desaturation during procedure.

In our study, none of the patients developed apnea, bradycardia or arrest. In our study, none of the patients developed emergence reactions, this may be due to young age of the sample size that ranged from 2 to 12 years with mid age of 5 years and these reactions, unpleasant dreams or hallucinations are mainly found to be transient and mild in children [12], however recovery agitation occurred in (2.4%) in study done by Green and his colleagues, 2011 [13].

Ketamine was found to increase heart rate nearly for all children more than that measured before injection in pre-sedation assessment, but it was accepted for their age.

Also, ketamine was found to increase blood pressure nearly for all children more than that measured before in the pre-sedation assessment, but most of them were accepted for their age. Hypertension occurred above-accepted level for age in 4 cases (4%), 2 of them was in group (4) who had high blood pressure in pre-sedation assessment before ketamine injection, one in group (1), this patient was previously diagnosed with ITP and received steroids, and one in group (2), this patient was Steroid Resistant Nephrotic Syndrome (SRNS), receiving steroids and the endoscopy was requested as a routine preparation of renal transplantation.

In all groups, there is no relation between ketamine dose and complications except in group (3), this could not be assessed as there is no complications occurred in this group.

One patient (1%) in the group (2) developed post-procedural vomiting, this female patient is SRNS, was receiving steroids, received ketamine at a dose of 2.5 mg/kg, had high blood pressure for her age during the procedure and her upper GI endoscopy revealed gastritis.

A randomised clinical trial suggested that the use of 0.01 mg/kg of atropine reduced hypersalivation and vomiting associated with IM ketamine; there are no data on this strategy when used with IV ketamine [14].

A study was done by Green et al., 2011 [13] which is different from our study as it was retrospective in 636 cases primarily by the intravenous (IV) route (98%), but our study is prospective study on 100 cases primarily on the intramuscular (IM) route (65%).

This study is similar to our study as it was done primarily for EGD (86%) and our study was done also primarily for EGD (76%).

Adverse effects of this study included transient laryngospasm (8.2%), vomiting (4.1%), recovery agitation (2.4%), partial airway obstruction (1.3%), apnea and respiratory depression (0.5%), and excessive salivation (0.3%) and this differs completely from our study as we had desaturation (13%), vomiting (1%) with no other adverse effects but it may be due to this study had nearly half (46%) the subjects had severe underlying illness (American Society of Anesthesiologists (ASA] class > or =3) and our study had exclusion criterion of any severe illness.

In this study, all instances of laryngospasm occurred during EGD (9.5% incidence), and the only independent predictor of laryngospasm in this sample was decreasing age. The incidence of laryngospasm was 13.9% in preschool-aged (< or = 6 years) children and was 3.6% in school-aged (> 6 years) children (difference 10.3%, 95% confidence intervals 5.5-14.9%). No dose relationship was noted with laryngospasm. This is similar to our study as desaturation occurred mainly in EGD (14%) and there is no relation between ketamine dose and desaturation but differs that no relations between age and desaturation in our study.

In Regional Hospital of Iquique, Iquique, Chile, over the past 24 years, with the authorization of the anesthesiology department, they have used ketamine in more than 900 paediatric endoscopic procedures. This experience has included upper endoscopy, intestinal biopsy, colonoscopy, percutaneous gastrostomy, ERCP, foreign-body removal, oesophagal dilation, and oesophagal sclerotherapy [15].

These indications are similar to our indications which included upper endoscopy for diagnosis and follow-up, colonoscopy (23%), foreign body removal (10%), oesophagal dilation (23%), and routine before Renal Transplantation.

Our data may be useful for planning larger studies required to determine whether laryngospasm or other adverse events occurs more or less frequently with ketamine than has been reported previously.

## References

[1] Schwarz SM, Lightdale JR, Liacouras CA (2007). Sedation and anesthesia in pediatric endoscopy: one size does not fit all. J Pediatr Gastroenterol Nutr.

[2] Lauven PM (1990). Pharmacology of drugs for conscious sedation. Scand J Gastroenterol.

[3] Deasy C, Babl FE (2010). Intravenous vs intramuscular ketamine for pediatric procedural sedation by emergency medicine specialists: a review. Paediatr Anaesth.

[4] National Institute on Drug Abuse (2001). NIDA Research Report Series, Hallucinogens and Dissociative Drugs.

[5] Roelofse JA (2010). The evolution of ketamine applications in children. Paediatr Anaesth.

[6] Gilger MA, Spearman RS, Dietrich CL, Spearman G, Wilsey MJ, Zayat MN (2004). Safety and effectiveness of ketamine as a sedative agent for pediatric GI endoscopy. Gastrointestinal Endoscopy.

[7] Green SM, Klooster M, Harris T, Lynch EL, Rothrock SG (2001). Ketamine sedation for pediatric gastroenterology procedures. Journal of Pediatric Gastroenterology and Nutrition.

[8] Reich DL, Silvay G (1989). Ketamine: an update on the first twenty-five years of clinical experience. Canadian Journal of Anaesthesia.

[9] Aggarwal A, Ganguly S, Anand VK, Patwari AK (1998). Efficacy and safety of intravenous ketamine for sedation and analgesia during pediatric endoscopic procedures. Indian Pediatrics.

[10] Spearman RS, Spearman G, Wilsey MJ, Gilger M (2000). Is Ketamine a “safe” sedative agent for pediatric endoscopy?. Journal of Pediatric Gastroenterology and Nutrition.

[11] Green SM, Johnson NE (1990). Ketamine sedation for pediatric procedures: Part 2, review and implications. Ann Emerg Med.

[12] Howes MC (2004). Ketamine for paediatric sedation/analgesia in the emergency department. Emerg Med J.

[13] Green SM, Roback MG, Kennedy RM, Krauss B (2011). Clinical Practice Guideline for Emergency Department Ketamine Dissociative Sedation: 2011 Update. Annals of Emergency Medicine.

[14] Heinz P, Geelhoed BC, Wee C, Pascoe EM (2006). Is atropine needed with ketamine sedation? A prospective, randomised double blind trial. Emerg Med J.

[15] Kirberg A, Sagredo R, Montalva G, Flores E (2005). Ketamine for pediatric endoscopic procedures and as a sedation complement for adult patients. Gastrointest Endosc.

